# Treatment of Cerebral Rheumatism by Hydrate of Chloral

**Published:** 1875-11

**Authors:** 


Treatment of Cerebral Rheumatism by Hydrate of Chloral—New Method. (From report of Academy of Sciences.)—M. Bouchut communicated the following note:

The displacement of acute articular rheumatism and its transfer to the membranes of the brain, called cerebral rheumatism or rheumatismal meningitis, is always attended with great danger.

Pathological anatomy and ophthalmoscopy prove that this complication of acute articular rheumatism is a form of meningitis. Examination of the membranes of the brane reveals a considerable venous stasis, with an opaline infiltration of the pia mater—caused by numerous lucocytes.

The ophthalmoscope, that permits us to follow in the fundus of the eye the development of the alterations going on in the cerebral substance and in the meninges, shows a serious infiltration of the papilla and of the adjoining portion of the retina, with dilatation of the retinal veins, which represent the similar alterations of the pia mater and of the brain.

The rheumatism of the brain manifests itself by delirium, more or less violent, terminating in coma and in asphyxia, often so rapid as to cause death in several hours.

In three cases of this nature the patients were cured by the hydrate of chloral taken by the mouth, in quantity of three to six grammes, in one or two doses, one rapidly following the other, so as to quickly calm the agitation under which such patients labor. —*Cincinnati Lancet and Observer.*

